# Novel Mechanisms of Herbal Therapies for Inhibiting HMGB1 Secretion or Action

**DOI:** 10.1155/2015/456305

**Published:** 2015-03-02

**Authors:** Andrew H. Wu, Li He, Wei Long, Qiuping Zhou, Shu Zhu, Ping Wang, Saijun Fan, Haichao Wang

**Affiliations:** ^1^Department of Emergency Medicine, North Shore University Hospital, Manhasset, NY 11030, USA; ^2^The Feinstein Institute for Medical Research, North Shore-LIJ Health System, 350 Community Drive, Manhasset, NY 11030, USA; ^3^Department of Ophthalmology, University of Alabama at Birmingham, Birmingham, AL 35294, USA; ^4^The Radiation Medicine Institute of Chinese Academy, Medical Sciences and Peking Union Medical College, Tianjin 300192, China

## Abstract

High mobility group box 1 (HMGB1) is an evolutionarily conserved protein and is constitutively expressed in virtually all types of cells. In response to microbial infections, HMGB1 is secreted from activated immune cells to orchestrate rigorous inflammatory responses. Here we review the distinct mechanisms by which several herbal components inhibit HMGB1 action or secretion, such as by modulating inflammasome activation, autophagic degradation, or endocytic uptake. In light of the reciprocal interactions between these cellular processes, it is possible to develop more effective combinational herbal therapies for the clinical management of inflammatory diseases.

## 1. Introduction

High mobility group box 1 (HMGB1), an evolutionarily conserved 30 kDa DNA-binding protein, is ubiquitously expressed in virtually all types of cells. Bearing two nuclear-localization sequences (NLS), HMGB1 is transported into the nucleus by the nuclear import complexes, thereby maintaining a large nuclear “pool” of preformed protein [1]. It carries two internal repeats of positively charged domains (“HMG boxes” known as “A box” and “B box”) in the N-terminus and a continuous stretch of negatively charged (aspartic and glutamic acid) residues in the C-terminus. These HMG boxes enable HMGB1 to bind chromosomal DNA and fulfill its nuclear functions in stabilizing nucleosomal structure and regulating gene expression [1]. The disruption of local expression of HMGB1 renders animals susceptible to infectious [2] or injurious insults [3, 4], reinforcing a beneficial role of intracellular HMGB1 in immunity against infection and injury [5].

In response to infections and injuries, however, HMGB1 is secreted from activated immune cells or passively released from injured cells. Excessive HMGB1 secretion/release adversely contributes to the pathogenesis of infection- and injury-elicited inflammatory diseases. For instance, in animal models of endotoxemia or sepsis (induced by cecal ligation and puncture, CLP), HMGB1-neutralizing antibodies improve survival [6] and rescue rodents from lethal sepsis even if given at 24 h after CLP [7, 8]. Similarly, HMGB1-specific antibodies are protective against ischemia/reperfusion [9–11], trauma [12, 13], chemical toxemia [14–16], atherosclerosis [17], gastric ulcer [18], and hyperoxia [19], supporting the pathogenic role of HMGB1 in injury-elicited inflammatory diseases. Furthermore, in animal models of rheumatoid arthritis, anti-HMGB1 agents confer significant protection against joint tissue edema [20–22], supporting a pathogenic role for HMGB1 in autoimmune diseases. The establishment of HMGB1 as a mediator of various inflammatory diseases has prompted the search for inhibitors that can attenuate HMGB1 secretion or action. In this review, we summarize the divergent mechanisms by which several herbal therapies effectively inhibit active HMGB1 secretion and action and hope to stimulate interests in developing novel HMGB1-targeting therapeutic strategies for the treatment of inflammatory diseases.

## 2. Regulation of HMGB1 Secretion

In response to microbial products (e.g., ds-RNA, CpG-DNA, and endotoxins) [6, 23], macrophages/monocytes secrete HMGB1 into the extracellular milieu in a delayed fashion. Lacking a leader peptide sequence, HMGB1 cannot be actively secreted through classical endoplasmic reticulum-Golgi exocytotic pathways [6]. Instead, it is first shuttled to cytoplasmic vesicles (“nucleus-to-cytoplasm translocation”) and subsequently secreted into the extracellular environment. The nucleus-to-cytoplasm translocation is regulated by posttranslational modifications (e.g., acetylation or phosphorylation) [24, 25] of the NLS [5, 26]. For instance, bacterial endotoxin or proinflammatory cytokines (e.g., IFNs) can activate the JAK/STAT1 signaling pathways and acetylate lysine residues within the NLS sites, leading to sequestration of HMGB1 into cytoplasmic vesicles [24, 27–29].

Subsequently, cytoplasmic HMGB1 is secreted into the extracellular space partly through caspase-1-mediated pyroptosis, a proinflammatory programmed cell death by which activated macrophages rapidly release large amounts of cellular contents (including HMGB1 and cytokines such as IL-1*β*) extracellularly. Indeed, pharmacological inhibition (with a broad-spectrum caspase inhibitor Z-VAD-FMK) or genetic disruption of caspase 1 uniformly reduces HMGB1 secretion [8, 30]. Specifically, the procaspase-1 forms a heteromeric protein complex with an adaptor protein (termed apoptosis-associated speck-like protein containing a CARD, ASC), a NOD-like receptor (NLR, e.g., NLRP1, NLRP3, and NLRC4), or a member of the PYHIN family. The resultant protein complex, termed the “inflammasome,” is responsible for cleaving procaspase-1 to generate caspase-1, which triggers inflammasome activation as well as pyroptosis [30]. Inflammasome activation occupies an essential role in the regulation of HMGB1 secretion [30, 31], because genetic disruption of key inflammasome components (e.g., caspase 1 or Nalp3) completely blocks LPS/ATP-induced HMGB1 secretion. Recently, the double-stranded RNA-activated protein kinase R (PKR) has been established as a key regulator of inflammasome activation and HMGB1 secretion [31]. Consistently, genetic disruption of PKR expression or pharmacological inhibition of PKR phosphorylation (with 2-aminopurine (2-AP) or 7-desacetoxy-6,7-dehydrogedunin (7DG)) markedly reduces inflammasome activation [31, 32], pyroptosis [31, 32], and HMGB1 secretion [31]. Thus, the LPS- or IFN-induced HMGB1 secretion is controlled not only by JAK/STAT-mediated acetylation and nuclear-cytoplasmic translocation, but also through PKR-mediated inflammasome activation and pyroptosis [5, 26].

## 3. Extracellular Role of HMGB1 as an Alarmin

**Figure 1:**
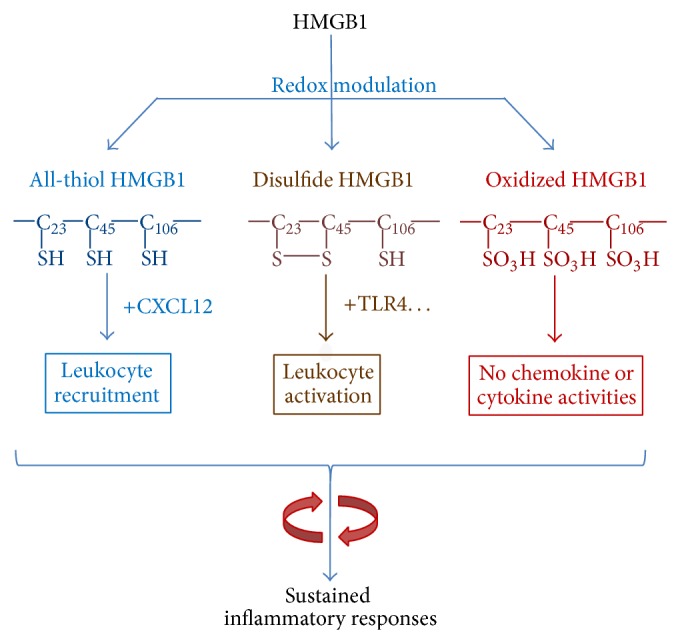
Extracellular HMGB1 as a proinflammatory cytokine/chemokine. The immunological activities of HMGB1 are modulated by the redox status in a divergent fashion, thereby facilitating leukocyte recruitment or activation, resulting in sustained inflammatory responses.

Once released, extracellular HMGB1 functions as an alarmin signal to alert, recruit, and activate immune cells. For instance, HMGB1 binds to various microbial products (e.g., CpG-DNA or LPS), thereby facilitating their recognition by respective receptors to augment inflammatory responses [33]. Harboring three cysteine residues (C23, C45, and C106) that are redox-sensitive, HMGB1 can be modified into three isoforms termed “HMGB1” (all-thiol form), “disulfide HMGB1” (partially oxidized), and oxidized HMGB1 (Figure 1) [34, 35]. The “all-thiol” HMGB1 binds to other chemokines (e.g., CXCL12) and stimulates leukocyte recruitment via the CXCR4 receptor [36]. The “disulfide” HMGB1 can activate immune cells to produce cytokines/chemokines via TLR4 or other receptors such as RAGE [33], TLR2, TLR4 [37–39], TLR9 [23, 33], cluster of differentiation 24 (CD24)/Siglec-10 [40], Mac-1 [41], thrombomodulin [42], or single transmembrane domain proteins (e.g., syndecans) [43]. Once fully oxidized, the HMGB1 is devoid of either chemokine or cytokine activities (Figure 1) [34, 35]. Thus, HMGB1 can function either as a chemokine to stimulate leukocyte migration [41, 44, 45] or as a cytokine to activate macrophages [37, 46, 47] and endothelial cells [48, 49] to produce more cytokines, chemokines, and adhesion molecules.

## 4. Distinct Mechanisms of Herbal Inhibition of HMGB1 Secretion or Action

**Figure 2:**
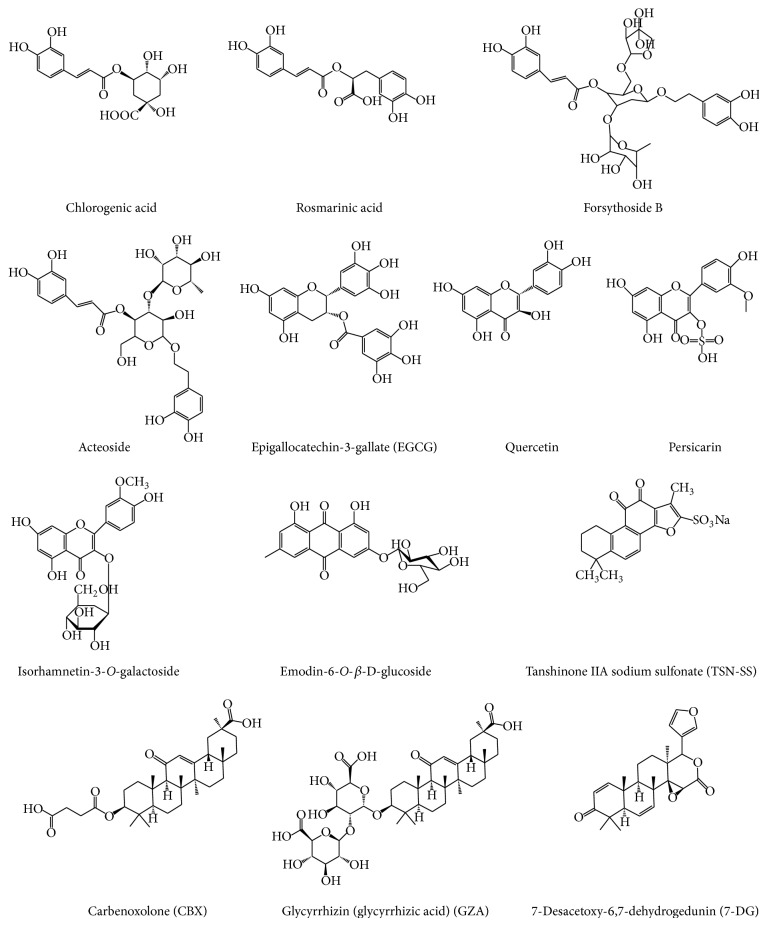
Chemical structures of HMGB1-inhibiting herbal components.

Recently, a number of herbal extracts (e.g., Danggui, Mung bean, and* Prunella vulgaris*) [50–52] and components (e.g., nicotine, EGCG, tanshinone, glycyrrhizin, chlorogenic acid, emodin-6-O-*β*-D-glucoside, rosmarinic acid, isorhamnetin-3-O-galactoside, persicarin, forsythoside B, chloroquine, acteroside, and shikonin) (Figure 2) [53–65] have been shown effective in inhibiting endotoxin-induced HMGB1 secretion. Here we compare the distinct mechanisms by which several herbal components effectively inhibit HMGB1 action or secretion.

### 4.1. Glycyrrhizin (GZA) Binds to HMGB1 to Inhibit Its Secretion or Action

**Figure 3:**
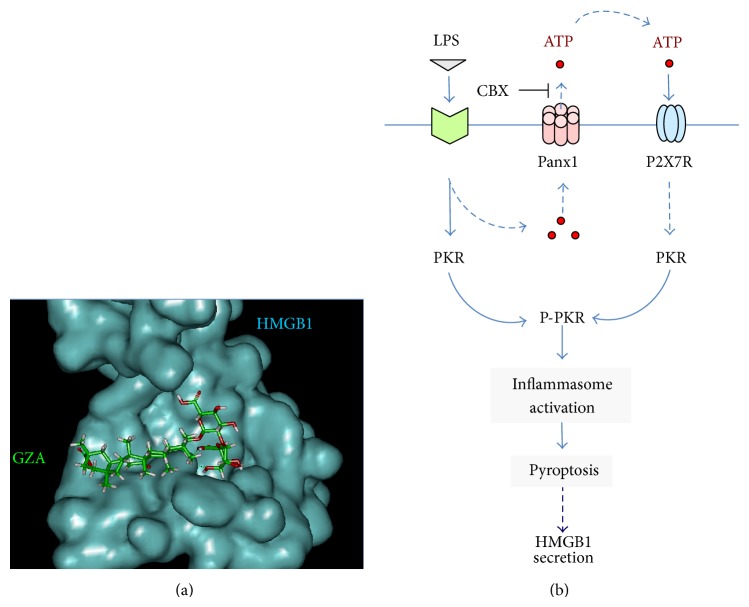
Divergent inhibition of HMGB1 action or secretion. (a) GZA binds to the shallow cave surface of HMGB1 boxes. Computer-assisted molecular docking of HMGB1 with GZA: the blue area represents surface of HMGB1 box A, whereas the chemical structure of GZA is shown in green. (b) CBX inhibits LPS-induced HMGB1 secretion by preventing PKR activation. Prolonged stimulation with crude LPS may lead to panx-1 hemichannel-mediated ATP efflux and upregulation of PKR expression. Extracellular ATP then binds to P2X7R and activates the ATP-gated P2X7R and panx-1 hemichannels, leading to PKR phosphorylation and subsequent inflammasome-dependent HMGB1 secretion. CBX may block LPS-induced ATP efflux through panx-1, thereby impairing ATP/P2X7R-mediated PKR activation and subsequent inflammasome-dependent HMGB1 secretion.

Gancao (radix glycyrrhizae, meaning “sweet root” in Greek or “licorice” in English) has been traditionally used in the clinical management of various inflammatory diseases including peptic ulcer, hepatitis, and pulmonary bronchitis for many centuries. Its anti-inflammatory properties are attributable to a major component, glycyrrhizin (GZA, Figure 2), which has been proven beneficial in animal models of hepatitis [66], hepatic ischemia/reperfusion (I/R) injury [67, 68], endotoxin- and acetaminophen-induced liver injury [69, 70], influenza [71], lung inflammation [72], intracerebral hemorrhage [73], cerebral I/R injury [74, 75], seizure [76], endotoxemia [56, 77], and colitis [78]. Sakamoto et al. first employed biochemical techniques and demonstrated that GZA directly interacted with HMGB1 to induce certain conformational changes that prevented DNA-binding [79]. Subsequently, Mollica et al. (2007) used nuclear magnetic resonance (NMR) and fluorescence techniques and confirmed that GZA indeed docked into the DNA-binding concaves of both HMGB1 boxes (Figure 3(a)) [80, 81]. In agreement with these findings, most GZA-mediated protective effects have been associated with the inhibition of HMGB1 release [56, 68, 75, 76] or cytokine/chemokine activities [56, 70, 73, 82].

### 4.2. Carbenoxolone (CBX) Prevents PKR Activation

The replacement of the glucuronic acid in GZA by succinic acid gives rise to a new compound, carbenoxolone (CBX, Figure 2), a drug previously prescribed for esophageal ulceration and inflammation [83]. Since its inception, CBX has been shown to dose-dependently inhibit a variety of biological activities including the gap junctions (50–100 *μ*M) and the panx-1 hemichannels (EC_50_ = 1–4 *μ*M) [84, 85]. Recently, we discovered that CBX also effectively inhibited LPS-induced HMGB1 secretion, with an estimated IC_50_ and IC_100_~5 *μ*M and 10 *μ*M, respectively [86]. However, it is unlikely that CBX inhibits the LPS-induced HMGB1 secretion through impairing the gap junctions, because macrophages do not form gap junctions, and the concentrations of CBX used to block gap junctions (i.e., 50–100 *μ*M) are much higher than those (i.e., 5–10 *μ*M) used to abrogate LPS-induced HMGB1 secretion [86].

The involvement of PKR in CBX-mediated inhibition of HMGB1 secretion is supported by several lines of evidence. First, ultrapure LPS (*free from contaminating bacterial proteins and nucleic acids*) fails to trigger HMGB1 secretion unless the initial LPS (10 *μ*g/mL) priming is accompanied by a second stimulus (e.g., ATP) [30, 31], which promotes PKR phosphorylation [31] and inflammasome activation [87–89]. Second, crude LPS* (containing trace amounts of bacterial proteins and nucleic acids*) triggers marked upregulation of PKR expression (>2-fold) and phosphorylation (>8-fold) and effectively induces HMGB1 secretion [6]. It is possible that the crude LPS may prime macrophages by upregulating PKR expression and simultaneously eliciting panx-1-mediated ATP release (Figure 3(b)). Extracellular ATP then binds and activates the purinergic P2X7 receptor (P2X7R) [90], which further elevates panx-1 hemichannel activity to induce feed-forwarding ATP release and subsequent PKR/inflammasome activation and HMGB1 secretion [87–89] (Figure 3(b)). This hypothesis is consistent with the finding that panx-1 physically interacts with both P2X7R and components of the NLRP3 inflammasome [91, 92]. It is also supported by our observations that both P2X7R antagonists (e.g., oxidized ATP or oATP) and panx-1 inhibitors (e.g., CBX) effectively inhibit LPS-induced dye uptake, PKR activation, and HMGB1 secretion (Figure 3(b)) [31, 93]. Consistently, CBX (10 *μ*M) has recently been proven effective in inhibiting the panx-1-mediated ATP release in response to hypoxia [94], sheer stress [95], and low oxygen tension [96] and blocking HMGB1 secretion by neurons during cortical spreading depression [97].

### 4.3. Epigallocatechin-3-Gallate (EGCG) Stimulates Autophagic HMGB1 Degradation

**Figure 4:**
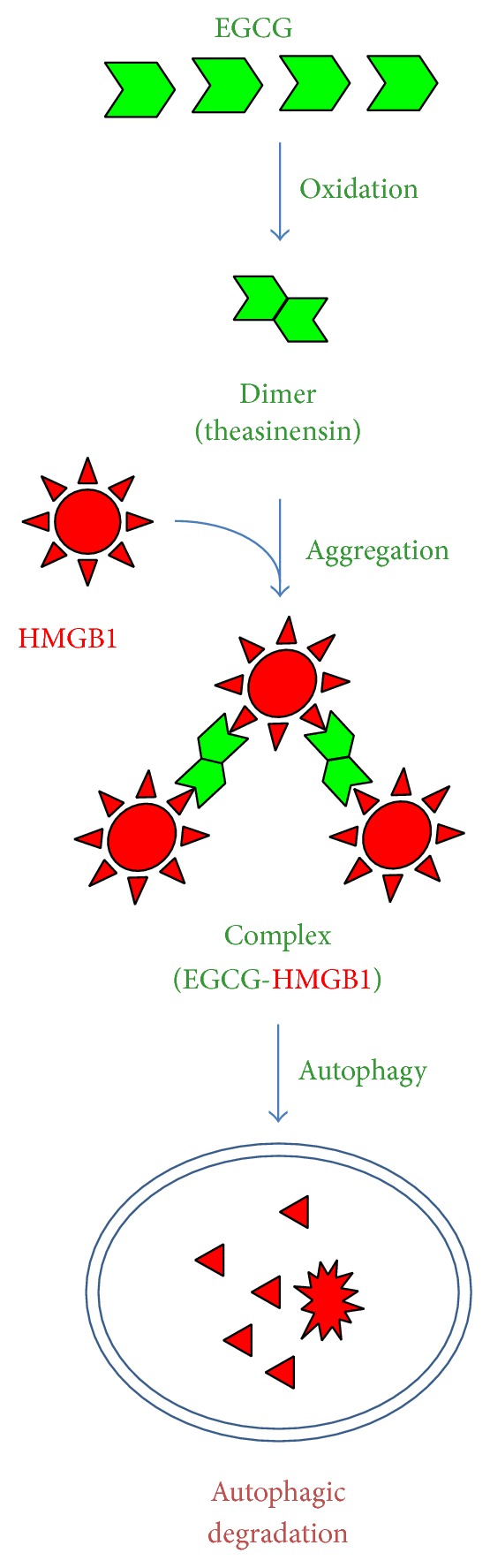
EGCG induces autophagic HMGB1 degradation. Green tea EGCG induces HMGB1 aggregation, thereby triggering autophagic HMGB1 degradation in macrophage cultures.

Green tea contains a class of biologically active polyphenols called catechins such as the epigallocatechin-3-gallate (EGCG). At relatively low concentrations (10–15 *μ*M), EGCG partially inhibits LPS-induced release of TNF and IL-12 but dramatically attenuates IL-6 and several chemokines (including MIP-1*α*, MIP-1*β*, MIP-2, RANTES, KC, MCP-1, and CXCL16) [54]. Similarly, EGCG dose-dependently abrogates LPS-induced HMGB1 secretion, with an estimated IC_50_ < 1.0 *μ*M [54]. Notably, significant inhibition of HMGB1 secretion is still achieved even when EGCG is added 2–6 h after LPS stimulation [54], suggesting EGCG as an effective HMGB1 inhibitor. It now appears that EGCG prevents the LPS-induced HMGB1 secretion strategically by destroying HMGB1 in the cytoplasm via a cellular degradation process, autophagy (self-eating) (Figure 4).

As an evolutionarily conserved cellular process for degrading damaged cytoplasmic macromolecules, autophagy begins with the formation of double-membraned structures, which elongate and engulf portions of the cytoplasm to form autophagosomes. Subsequently, autophagosomes fuse with lysosomes to form degradative autophagolysosomes, where the engulfed contents are degraded by acidic lysosomal hydrolases. Indeed, EGCG can be trafficked into autophagosomes within 6 h and then destined to the lysosomal-associated membrane protein 2- (LAMP2-) containing autophagolysosomes within 16 h [98]. Meanwhile, EGCG conjugates to cytoplasmic HMGB1, leading to the formation of EGCG-HMGB1 complexes (dimmer, trimmer, tetramer, and oligomer) (Figure 4) [98]. This is consistent with previous findings that EGCG may conjugate to proteins either covalently with the free thiol group of cysteine residues [99] or noncovalently via hydrogen bonding, aromatic stacking, or hydrophobic interactions [100]. Because these large EGCG-HMGB1 complexes cannot physically pass through the narrow pore of the proteasome barrel of the ubiquitin-proteasome pathway, they trigger the autophagic degradation process. Consistently, at the concentrations effective for inhibiting HMGB1 secretion, EGCG dramatically enhances the formation of autophagosomes [98]. In contrast, the coaddition of autophagy inhibitors (e.g., 3-methyladenine) impairs EGCG-mediated inhibition of HMGB1 secretion, thereby leading to a dramatic accumulation of HMGB1 aggregates in macrophages. Recently, EGCG has also been proven effective in stimulating autophagy in other cell types including breast cancer cells [98], hepatocytes [101], retinal pigment epithelial cells [102], and vascular endothelial cells [103]. Given the possibility that HMGB1 interacts with autophagy regulators (e.g., beclin-1) in the cytoplasm [104, 105], it will be important to investigate whether HMGB1 occupies a critical role in EGCG-mediated autophagy. This is relevant because recent studies indicate that bacterial endotoxin induces significantly less autophagy in HMGB1-deficient macrophages [2].

### 4.4. Tanshinone IIA Sodium Sulfonate (TSN-SS) Stimulates Endocytic HMGB1 Uptake

Danshen is a medicinal herb that contains several red pigments including tanshinones I, II, and IV and cryptotanshinone, which exhibit various anti-inflammatory properties. Accounting for 5-6% of the total dry weight of Danshen root, tanshinone IIA dose-dependently attenuates LPS-induced HMGB1 secretion, with an estimated IC_50_ < 25 *μ*M. However, its poor water solubility may adversely affect the bioavailability and therapeutic efficacy of tanshinone IIA [55], thereby prompting the exploration of water-soluble derivatives as more effective HMGB1 inhibitors. One such compound, tanshinone IIA sodium sulfonate (TSN-SS), dose-dependently inhibits LPS-induced HMGB1 secretion with an estimated IC_50_ < 10 *μ*M. At the doses that completely prevent HMGB1 secretion, TSN-SS does not affect endotoxin-induced release of most other cytokines and chemokines (such as IL-6, IL-12p40/p70, KC, MCP-1, MIP-1*α*, MIP-2, and TNF), indicating a selectivity for TSN-SS in inhibiting HMGB1 secretion.

**Figure 5:**
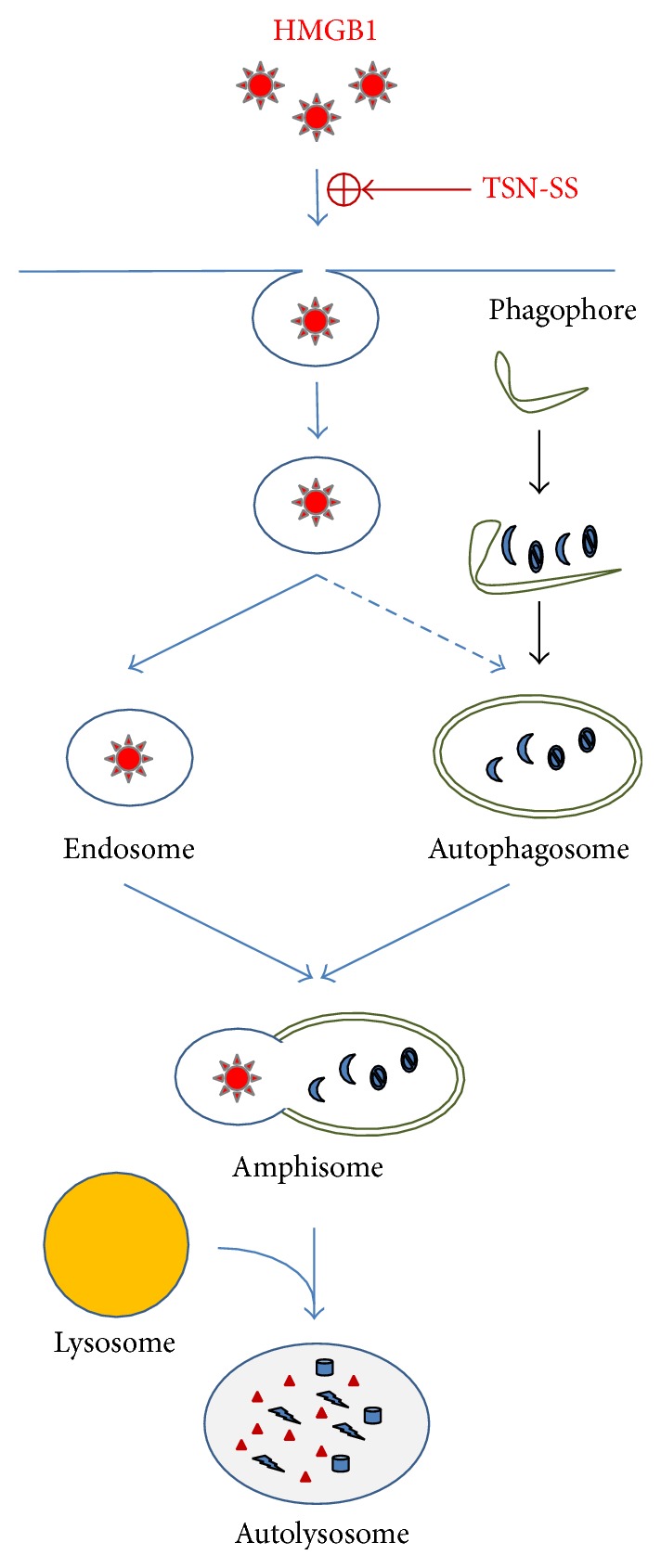
TSN-SS stimulates endocytic HMGB1 uptake. TSN-SS facilitated internalization of exogenous HMGB1 possibly via clathrin- and caveolin-dependent endocytosis into cytoplasmic vesicles that eventually mature into endosomes. Consequently, it likely triggers another cellular degradation process, autophagy, during which cytoplasmic macromolecules are engulfed by double-membraned cytoplasmic vesicles termed autophagosomes. Subsequently, these HMGB1-containing endosomes could be fused with other cytoplasmic vesicles (such as autophagosomes) to form amphisomes, where the internalized HMGB1 was likely degraded via a lysosome-dependent mechanism.

Unlike EGCG, TSN-SS itself is unable to stimulate autophagic HMGB1 degradation [55] but instead induces the internalization of exogenous HMGB1 into cytoplasmic vesicles possibly through clathrin- and caveolin-dependent endocytosis (Figure 5) [106]. Indeed, specific inhibitors for both clathrin- (e.g., chlorpromazine) and caveolin-dependent (e.g., nystatin and indomethacin) endocytosis uniformly attenuate the TSN-SS-mediated HMGB1 uptake. Surprisingly, the depletion of several HMGB1 receptors (e.g., TLR2, TLR4, or RAGE) does not impair TSN-SS-mediated enhancement of HMGB1 uptake, suggesting that other HMGB1-binding cell surface proteins (such as Mac-1, thrombomodulin, or syndecan) may be required for the TSN-SS-mediated HMGB1 uptake.

Given the regulatory role of HMGB1 in autophagy [2, 104, 105], the TSN-SS-mediated HMGB1 endocytosis may be linked to autophagy (Figure 5). When occurring simultaneously, endocytosis and autophagy can converge on a common lysosome-dependent pathway, leading to eventual HMGB1 degradation. Specifically, endosomes fuse with autophagosomes to form amphisomes [107, 108], which then merge with lysosomes to form autolysosomes, where the amphisome contents are digested by lysosomal enzymes [109]. In the presence of TSN-SS, exogenous HMGB1 was detected in increased number of larger cytoplasmic vesicles that colocalized with autophagy- (LC3-positive) punctate structures, suggesting that HMGB1-containing endosomes may have been fused with autophagosomes to form amphisomes. The internalized HMGB1 is then possibly degraded via the lysosome-dependent pathway, because bafilomycin A1, a specific inhibitor of autophagosome-lysosome fusion, prevents the degradation of LC3-II and exogenous HMGB1. Taken together, these results suggest that TSN-SS facilitates endocytosis of exogenous HMGB1, leading to subsequent HMGB1 degradation via a lysosome-dependent pathway (Figure 5). Notably, even when given several hours after the endotoxin stimulation, TSN-SS still effectively blocks HMGB1 secretion. It is thus possible to strategically administer TSN-SS in a delayed fashion to pharmacologically “recycle” injurious proinflammatory mediators (such as HMGB1) back to innate immune cells. TSN-SS has already been used in China as a medicine for patients with cardiovascular disorders, and its capacity to facilitate endocytic HMGB1 uptake by professional phagocytes may provide basis for the treatment of both infection- and injury-elicited inflammatory diseases [26].

## 5. Therapeutic Efficacy of HMGB1-Inhibiting Herbal Components

Given the capacity of various herbal components in preventing endotoxin-induced HMGB1 secretion, we explored their efficacy in animal models of CLP-induced sepsis. Considering the late and prolonged kinetics of HMGB1 accumulation in experimental sepsis [7], the first dose of HMGB1 inhibitors was given in a delayed fashion, 24 h after the onset of sepsis. Repetitive intraperitoneal administration of EGCG [54], TSN-SS [55], or CBX [86], at 24, 48, and 72 h after CLP, significantly increased animal survival rates. When given orally, EGCG still rescued mice from lethal sepsis, significantly increasing animal survival rates from 16% to 44% [98]. Intriguingly, we found that EGCG facilitated bacterial elimination in selective organs (e.g., the liver and lung) in an animal model of sepsis [110]. Importantly, these herbal components have also been proven beneficial in other models of inflammation such as ischemia [68, 111–117], trauma [118–120], crush injury [121], radiation [122, 123], and chemical toxemia [124, 125]. It is not yet known whether these protective effects are also associated with inhibition of HMGB1 release or chemokine/cytokine activities.

Recently, a herbal remedy consisting of five herbs (Danggui, Danshen, Honghua, Chuanxiong, and Chishao) has been developed in China for treating septic patients. This combinational therapy, termed “Xuebijing,” has been proven to be protective experimentally in animal model of sepsis [126] or clinically in patients with sepsis [127, 128]. In light of the distinct but occasionally complementary mechanisms of herbal inhibition of HMGB1 release or action, combinational therapy with multiple herbs might result in an improved therapeutic efficacy. For instance, the induction of autophagy by EGCG may provide a negative feedback regulation of inflammasome activation at multiple levels such as by eliminating damaged mitochondria (to prevent mitochondrial DNA release) [129], removing active inflammasomes [129, 130], and destroying cytoplasmic HMGB1 [98]. It is thus important to test whether a better protection could be achieved by combinational therapy with several HMGB1 inhibitors that divergently modulate autophagy (e.g., EGCG) and inflammasome (e.g., CBX). These important studies may pave the road for future clinical studies to explore the therapeutic potential of additional herbal cocktail for the treatment of sepsis and other inflammatory diseases.

## 6. Conclusions

HMGB1 is secreted by activated macrophages/monocytes through complex mechanisms including PKR-dependent inflammasome activation and pyroptosis. A growing number of herbal components have been proven to be effective in inhibiting endotoxin-induced HMGB1 secretion through divergently distinct mechanisms such as preventing PKR/inflammasome activation, stimulating HMGB1 autophagic degradation, and enhancing endocytic HMGB1 uptake. In light of the intricate relationship between endocytosis, autophagy, and inflammasome activation, it is important to test whether a better protection could be achieved by combinational therapy with several anti-HMGB1 agents.
